# An ensemble deep learning models approach using image analysis for cotton crop classification in AI-enabled smart agriculture

**DOI:** 10.1186/s13007-024-01228-w

**Published:** 2024-07-14

**Authors:** Muhammad Farrukh Shahid, Tariq J. S. Khanzada, Muhammad Ahtisham Aslam, Shehroz Hussain, Souad Ahmad Baowidan, Rehab Bahaaddin Ashari

**Affiliations:** 1 FAST School of Computing, National University of Computer & Emerging Sciences, Karachi, 75030 Pakistan; 2 Department of Information Systems, Faculty of Computing and Information Technology, King Abdulaziz University, Jeddah, 21589 Saudi Arabia; 3 Information Technology Department, King Abdulaziz University, Jeddah, 21589 Saudi Arabia; 4 Fraunhofer FOKUS, Kaiserin-Augusta-Alle 31 Berlin, 10589 Germany; 5 Computer Systems Engineering Department, Mehran University of Engineering and Technology, Jamshoro, 76062 Pakistan

**Keywords:** Agriculture, Cotton crops, Artificial intelligence, Deep learning, Transfer learning, CWT, FFT, Crop monitoring; cotton plants, Deep ensemble learning

## Abstract

**Background:**

Agriculture is one of the most crucial assets of any country, as it brings prosperity by alleviating poverty, food shortages, unemployment, and economic instability. The entire process of agriculture comprises many sectors, such as crop cultivation, water irrigation, the supply chain, and many more. During the cultivation process, the plant is exposed to many challenges, among which pesticide attacks and disease in the plant are the main threats. Diseases affect yield production, which affects the country’s economy. Over the past decade, there have been significant advancements in agriculture; nevertheless, a substantial portion of crop yields continues to be compromised by diseases and pests. Early detection and prevention are crucial for successful crop management.

**Methods:**

To address this, we propose a framework that utilizes state-of-the-art computer vision (CV) and artificial intelligence (AI) techniques, specifically deep learning (DL), for detecting healthy and unhealthy cotton plants. Our approach combines DL with feature extraction methods such as continuous wavelet transform (CWT) and fast Fourier transform (FFT). The detection process involved employing pre-trained models such as AlexNet, GoogLeNet, InceptionV3, and VGG-19. Implemented models performance was analysed based on metrics such as accuracy, precision, recall, F1-Score, and Confusion matrices. Moreover, the proposed framework employed ensemble learning framework which uses averaging method to fuse the classification score of individual DL model, thereby improving the overall classification accuracy.

**Results:**

During the training process, the framework achieved better performance when features extracted from CWT were used as inputs to the DL model compared to features extracted from FFT. Among the learning models, GoogleNet obtained a remarkable accuracy of 93.4% and a notable F1-score of 0.953 when trained on features extracted by CWT in comparison to FFT-extracted features. It was closely followed by AlexNet and InceptionV3 with an accuracy of 93.4% and 91.8% respectively. To further improve the classification accuracy, ensemble learning framework achieved 98.4% on the features extracted from CWT as compared to feature extracted from FFT.

**Conclusion:**

The results show that the features extracted as scalograms more accurately detect each plant condition using DL models, facilitating the early detection of diseases in cotton plants. This early detection leads to better yield and profit which positively affects the economy.

## Introduction

Agriculture is one of the most prominent and significant industries around the globe. Major economies such as India, Russia, and China are among the major agricultural producers [1]. It also attributes to countries like Somalia and Liberia’s major portion of gross domestic product (GDP). This proves that the purpose of agriculture is more than just a feeding source in today’s world. Various factors, including weeds, pests, and diseases, contribute to the loss of crop production [2, 3]. Cotton [3] is an important economic crop that contributes to the production of natural fiber. The cotton crop makes a substantial contribution by producing textiles. It allows the textile industry to expand. Plant protection [4] is crucial in cotton production, in addition to other considerations. Mostly these diseases initially affect the leaves, ultimately resulting in the death of the plant. Therefore, plant leaves play a crucial role as a source for detecting plant diseases.

Several under-developed countries are among the major cotton producers in the world, such as Pakistan; it is the world’s seventh largest fabric producer, and cotton accounts for 10% of national GDP, compared to 18.9% of the nation’s total agriculture sector [5]. Surprisingly, the cotton crop product chain employs 50% of all industrial labor and accounts for more than 60% of total exports [6]. Cotton plays a critical role in boosting the country’s agricultural-based economic growth. Surprisingly, the cotton crop product chain employs 50% of all industrial labor and accounts for more than 60% of total exports [6]. According to the data, Pakistan should ideally achieve cotton self-sufficiency. However, the reality is that it has been importing raw cotton in recent years. Specifically, Pakistan has not exported cotton since 2010, primarily due to the declining trend in cotton production. Pest infestation and diseases attack such as cotton leaf curl virus disease, frequent pink bollworm attacks, and whitefly infestation are some of the primary causes of crop production kg per hectare and area loss [7, 8]. Cotton production utilizes 25% of global insecticide and 10% of pesticide use, making it the most pesticide-intensive crop. In 2017, the average production was 753 kg per hectare, but by 2021, it had dropped to 445 kg per hectare [9]. This leads to the economic collapse of both the farmer and the country. Additionally, the combination of limited resources, a large population, and an underdeveloped agricultural infrastructure has negatively impacted both the input and output of cotton in Pakistan. The tabular depiction of the production of cotton in Pakistan from 2016–2021 is depicted in Table 1 [10]. Therefore, there is an urgent need for affordable agricultural technology and its widespread implementation to address these challenges and establish a positive balance between cotton input and output. The best practices to mitigate crop loss involve prevention, early detection, and the effective management of plant diseases. A quick and comprehensive diagnostic test that automatically measures the severity of the disease can help to limit losses [11]. Many developing countries right now diagnose diseases through visual observation [12], which necessitates agricultural experts with extensive field experience. However, individual farmers provide 80% of the world’s food [13], and most farmers struggle to correctly identify crop disease kinds. Nevertheless, the detection of disease in plants in a large farm field is a very difficult and time-consuming task that requires expertise [14]. This visual inspection is also prone to biases and optical errors. Various emerging techniques and methods have been applied to cater to this challenge, including Precision Agriculture [15] and AI [16].

Computer vision-based detection and identification is easier, more accurate, and cheaper [17]. Its fundamental concept is to replicate how humans see and understand their surroundings and then translate this understanding into computer models that computers can use. Various industries have leveraged its capabilities for precise and critical functions. These sectors encompass retail and manufacturing, supply chain management, surveillance, and security, as well as agriculture. This can be accomplished using a variety of techniques and approaches, including linear regression, logistic regression, support vector machines (SVM), random forest, clustering, Gaussian models, K-nearest neighbors (KNN), Naive Bayes (NB), and decision trees (DT). Implementing a data-driven approach enhances the reliability and cost-effectiveness of farming through informed decision-making. This involves optimizing resource utilization, including labor and pesticides. Several image processing and machine learning algorithms have been applied in the field of agriculture, especially for the detection of diseases. The author in [18] classified the leaves of 32 species of plants using Random Forest (RF) and CNN classifier models with an accuracy of 97.3%. Li et al. [19] performed a classification of apple leaf diseases by extracting the features such as colors, shape, and texture. These features were then trained on a neural network achieving an accuracy of 92.6%. Due to the variation in the effects of diseases and their symptoms, the detection of a specific disease is a troublesome task. Cargo and Smith [20] in their work employed image-processing techniques to detect cotton disease automatically. Upon digitally capturing an image of a target, various image processing methods can be employed to extract features from it. The significance of each feature is determined by the specific patterns to be emphasized in the image. Patterns represent distinctive features within an image. The benefit of categorizing images based on feature analysis is that patterns remain stable even when the fundamental conditions are altered. The background noise in real-world environments can impact the effectiveness of the applied color model in various ways. The author in [21] transformed the electroencephalography (EEG) signal data using bandpass filtering, this resulted in better performance than the competing algorithms. The wavelet transform has been widely used in different fields to efficiently break down noisy signals and capture nonstationary features [22]. They highlight a signal’s frequency content over time intervals and visualise how frequencies change over time, exposing transient occurrences and frequency shifts within a signal. Dual-tree complex wavelet transformations (DT-CWT) have been proposed for tasks such as image denoising, fault identification, medical science, and recognizing EEG signals [23, 24]. The effectiveness of a pattern recognition system is influenced not only by the classifier but also by the depth of information from which the system can distinguish itself [25]. The author in [26] employed the stepwise discriminant method and Principal Component Analysis (PCA), specifically the Bayesian discriminant method, to extract 18 characteristic parameters, encompassing color, texture, and shape information, from images of tomato leaf spots. To extract distinctive characteristics and build a discriminant model, the PCA and Fisher discriminate method (Fisher discriminate method) were utilised. Precision was 94.71 percent and 98.32 percent, respectively. Moreover, with the advent of computer vision using Deep learning techniques and state-of-the-art image processing, the detection of the disease has become more accurate and effective [27]. One major drawback of the use of deep learning models is that they require massive amounts of data for training. Currently, available datasets are either not adequate or very small which cannot be used for critical decision making. Transfer learning entails fine-tuning pre-trained Convolutional Neural Networks (CNNs) by retraining them with smaller datasets exhibiting a distribution different from the larger datasets originally used to train the network from the ground up [28]. Transfer learning stands out as the most effective approach to boost the dependability of CNN classifiers in detecting plant leaf diseases. Undoubtedly, the effectiveness is evident when utilizing CNN models pre-trained on the ImageNet dataset and retraining them specifically for leaf disease detection. Consequently, the integration of deep learning with transfer learning offers an innovative solution to address the limitation of insufficient plant disease data. DL in recent years has also been used with visualization techniques for better results and a clear understanding of the disease in review. The work in [29], for example, diagnosed 13 distinct types of plant diseases using CaffeNet CNN architecture and achieved a CA of 96.30%, which was superior to earlier approaches such as SVM. Several filters were also utilised to display illness areas. The authors in [30] used the freely available PlantVillage dataset with the CNN architecture of AlexNet and GoogLeNet. The unique aspect of this research was the comparison of two well-known CNN architectures and the effect of three scenarios (color, grayscale, and segmented) on the evaluation of performance indicators. GoogLeNet was found to have outperformed by AlexNet. At present, deep learning architectures are surpassing shallow or conventional models in terms of performance. Deep ensemble learning models combine the benefits of both deep learning models and ensemble learning, resulting in improved generalization performance for the final model. Individual models’ key problems are overfitting, local minima, unknown errors, and divergence [31]. The study in [32] introduced a medical image classification model based on integrated learning. This approach involves the integration of MobileNetV2 and DenseNet 169 architectures as feature extraction backbone networks, resulting in improved performance in the medical image classification task.

**Table 1:** Cotton production statistics, observed fields 2016–2021

	Area		Production		Yield	
Year	Hectare (in thousands)	% Change	Bales (in thousands)	% Change	(kg/Hec)	% Change
2016–17	2489	–	10,671	–	729	–
2017–18	2700	8.5	11,946	11.9	753	3.3
2018–19	2373	– 12.1	9861	– 17.5	707	– 6.1
2019–20	2517	6.1	9148	– 7.2	618	– 12.6
2020–21 (Provisional)	2079	– 17.4	7064	– 22.8	578	– 6.5

Against the backdrop of declining statistics in cotton production over the past few years in Pakistan. The following questions are addressed in our study:

- Can we implement computer vision and artificial intelligence to address problems faced by cotton industry?
- What are the capabilities and limitations of CWT and FFT in analyzing images of cotton leaves?
- How effective are deep learning models when applied to detect the condition of cotton leaves, using features extracted from CWT and FFT?
- Can a deep ensemble learning approach improve the accuracy of leaf condition detection?

**Fig. 1:**
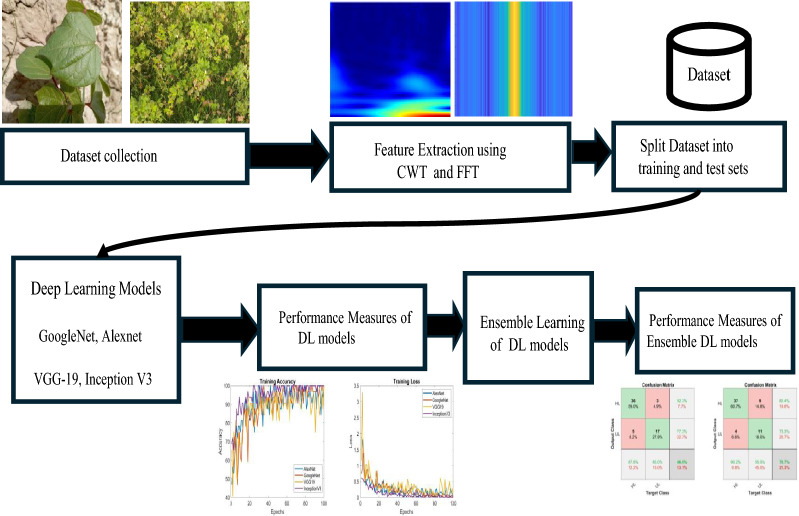
Flow diagram of the proposed work

In our study, we introduced a method for classifying healthy and unhealthy cotton plants through the application of deep learning techniques. Collection of the dataset in three phases from the cotton field located in the region under observation was done to acquire a generalized dataset. We employed transfer learning utilizing a dataset comprised of images obtained from the local field. Before feeding the images into CNN, we utilized spatial-temporal techniques, specifically CWT and FFT. Their ability to detect minor variations in leaf texture and spectral reflectance induced by diseases is enhanced by their ability to localise frequency changes in time and place. This can detect infections even before visual signs show, giving disease control a critical advantage. The classification process involved the utilization of four CNN models: AlexNet, GoogLeNet, InceptionV3, and VGG-19. An ensemble learning approach was introduced to tackle the issue of model bias and enhance classification accuracy. This involved combining the predictions of the individual CNN models. The ensemble model leverages the strengths of each base model, thereby achieving a more robust and accurate classification of healthy and unhealthy cotton plants. An evaluation was conducted, and the results demonstrated that the proposed method is effective for distinguishing between healthy and unhealthy cotton plants. Subsequently, the main contributions of the paper are also shown in Fig. 1 as follows:

- Investigate the problems associated with cotton crops.
- Feature extraction and analysis using CWT and FFT on the collected cotton plant dataset.
- Leveraging deep learning models to achieve classification task.
- Implementing a deep ensemble learning approach by combining deep learning models to enhance overall performance.
- Evaluate and demonstrate the effectiveness of the DL models and ensemble of DL models in terms of processing time and memory consumption.
- Comparative analysis of CWT and FFT-based implementations in terms of accuracy, precision, recall, and F1-score.

## Literature review

**Fig. 2:**
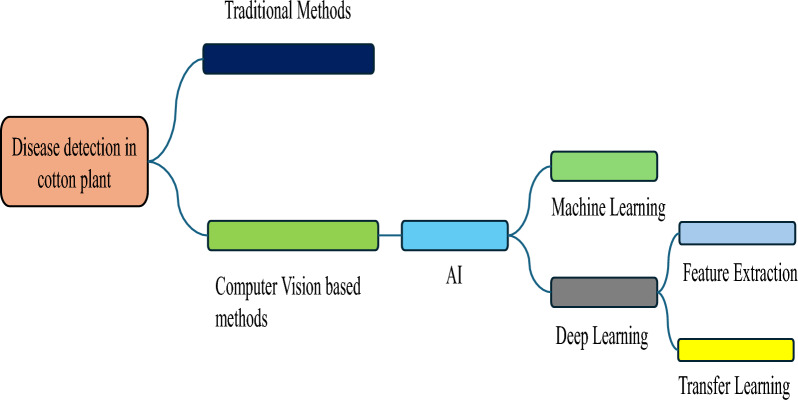
Plant disease detection state of the art methods overview

The introduction and integration of cutting-edge technologies and techniques like AI and the Internet of Things (IoT) have elevated the standards of agriculture. Currently, sustainable and precision agriculture has garnered global attention and popularity, due to its impactful outcomes on crop yield and profitability. In this context, a hybrid model was proposed by Zhan et al. [33] to extract information relevant to the leaves using CNN and Deep Convolutional Networks. Red, green, and blue (RGB) colors identify and classify leaf disease in cotton plants using a three-channel CNN model in [34]. Through a comprehensive survey of the literature, the author in [35] presents the current state-of-the-art in disease identification and classification from plant leaves using computer vision and soft computing approaches. It provides insights into the concepts, applications, and theories driving advancements in this field, with detailed discussions on various outcomes achieved by these methodologies. In [36] author uses different deep learning models of CNN with augmentation to classify the infected and healthy leaf of the citrus plant and deploy it to the platform as a service (PaaS). The dataset was divided into five class one for healthy leaves and the other 4 are of unhealthy leaves. 98% precision and recall were achieved along with F1 score of 99%.The authors in [37] employed K-mean clustering for dataset segmentation and detection was done through image processing algorithms. Approximately 500 distinct images showcasing diseases were gathered from the rice field for identification and classification. The processing was carried out using a CNN model [38]. In [39] author developed and implemented a system for tea leaf that predicts the real-time detection of diseased leaves. The models were evaluated using K-Fold cross-validation methods. The system outperforms the DCNN and achieves excellent accuracy. Yang et al. [40] used deep learning techniques for disease detection on leaves of mango plants. Five different diseases were identified using more than 600 images for training. An accuracy of 98.6% was achieved by the authors. The authors in [41] potatoes diseases were detected early using transfer learning of deep learning models, the models were trained on a public dataset that is available on Kaggle, this study highlights the significant improvement in result by just add more layers to the original architecture, among the different models DenseNet outperformed all other models that are discussed in the paper. In [42], detecting the diseases in banana plants. Multiple experiments were performed using different training models such as color and grayscale images and data split ratios. The work in [43] performed the classification of normal and abnormal images using a neural network and achieved an accuracy of 90%. Transfer learning was used along with the Mask-RCNN model to identify diseases in cotton leaves, with a 94% accuracy. NasNetlarge, VGG-19, DenseNet-121, ResNet-50, VGG-16, inceptionresnetv2, Xception, and inceptionv3 models were all fine-tuned for categorization [44]. The highest accuracy of 98.77% was attained by DenseNet-121. The author in [45] employed a genetic algorithm for the detection and classification of leaf diseases. Leveraging transfer learning with EfficientNet B7 and logistic regression the authors in [46] achieve a remarkable 98.7% accuracy in identifying leaf blight, black rot, powdery mildew, and black measles. Through comprehensive analysis, an effective classifier for practical application is proposed, demonstrating superior performance compared to existing methods. Zhu et al. [47], utilized thermal imaging technology for plant disease identification. One of the most important pre-processing stages for classification and identification processes is image segmentation. The authors proposed that thermal images provide better intuition and a wider detection range. For the detection of healthy and diseased wheat crop leaves, various image processing techniques, including feature extraction, image segmentation, shape features, texture features, and color attributes, were employed [48]. To expand the dataset size, a data augmentation technique based on a Generative Adversarial Network (GAN) was used. The dataset was then trained in CNN architecture achieving an accuracy of 98.70 [49]. Investigation of heart sound was done by the authors in [50] using a time-frequency representation. They concluded that the CWT provided the best representation of the time-frequency information in heart sounds when compared to Wigner distribution, short-time Fourier transform (STFT), and CWT. The authors in [51] proposed two novel algorithms, Image Preprocessing and Transformation Algorithm (IPTA) and Image Masking and REC-based Hybrid Segmentation Algorithm (IMHSA) to address the limited dataset and overfitting challenge. IPTA adaptively transforms original images into augmented ones, while IMHSA segments RGB images. A novel CNN model is trained on datasets before and after IPTA application, demonstrating significant improvement in accuracy, and solving the overfitting issue. Li et al. [52] proposed that feature vectors derived by FFT and wavelet transform can be used to identify and detect speed-up and speed-down defects in rotary machinery. The study in [53] introduces a deep convolutional neural network approach based on the Inception V4 architecture to identify weed density in soybean crop fields using the Crop Weed Field Image Dataset (CFWID). By utilizing RGB images and employing data cleaning techniques to remove background and foreground vegetation, the model effectively identifies weed-density areas. The author in [54] utilized a deep ensemble neural network for detecting plant leaf diseases, aiming to address the challenges associated with model bias and prediction errors. With limited computational resources, pre-trained models were employed in combination with an ensemble framework to reduce false positives and false negatives. To tackle the limitations associated with a single model, this study suggests employing ensemble learning by integrating multiple contemporary classification frameworks, including ResNet [55] and DenseNet [56]. The bagging ensemble learning algorithm is applied to extract models in each training iteration, marking the first instance of utilizing ensemble learning for the identification of wheat rust. Furthermore, the comparison of our work with existing related literature is tabulated in Table 2. The literature review strongly indicates that the application of computer vision techniques significantly improves yield and crop management. In our proposed framework, we leverage computer vision models through transfer learning and image analysis methods, namely CWT and FFT. To the best of our knowledge, this represents the first effort in utilizing these techniques for disease detection in plants, with a specific focus on cotton.  Figure 2 depicts an overview of state of the art disease detection method.

**Table 2:** Comparison of related works with the proposed work

Study	Year	Learning algorithm used	Dataset	Crop type	Training approach	Image analysis	Best accuracy
[57]	2020	S-CNN	PlantVillage Subset (15,817 Images)	Tomato	Image Segmentation	(X)	98.60%
[58]	2019	UNet + InceptionV3	Crop Disease AI Challenger 2018 (40,000 Images)	Multiple	Tune hyper-parameter	(X)	87.45%
[59]	2019	Three Channel CNN AlexNet	PlantVillage Subset (15,817 Images)	Tomato	Custom Layers	(X)	91.15%
[60]	2019	GoogLeNet	PDDB Embrapa (46,409 Images)	Multiple	Transfer Learning	(X)	94%
[61]	2019	ResNet-34	Collected Dataset(Field) (6267 Images)	Corn	Tune hyper-parameter	(X)	95.10%
[62]	2018	InceptionV3, MobileNet	PlantVillage Dataset (54.306 Images)	Multiple	Data Augmentation	(X)	88.6% 92.0%
[63]	2018	AlexNet	Collected Dataset(Field) (1184 Images)	Cucumber	Image Segmentation & Data Augmentation	(X)	93.40%
[64]	2020	ResNet, Xception	PlantVillage Dataset (4281 Images)	Tomato	Transfer Learning	(X)	99.37% 99.95%
[65]	2020	DNN	Collected Dataset(Field) (650 Images)	Rice	Jaya Optimized Algoritm	(X)	98.90%
[66]	2020	DCNN	Collected Dataset(Field) (1800 Images )	Rice	Automatic Selection	(X)	90.90%
[67]	2021	GoogLeNet, ResNet50	Collected Dataset(Field) (60,659 Images)	Cotton	Image Segmentation	(X)	95.2% 95.1%
[68]	2022	VGG-19	Rice Leafs 5 (2400 Images)	Rice	Fine-Tuning	(X)	96.08%
[69]	2022	MobileNet	Tomato Leaf (5452 Images)	Tomato	Filtering & Thresholding	(X)	98.7%
[70]	2023	Custom Model	CollectedDataset(Field)	Cotton	Transfer Learning	(X)	99.39%
[71]	2023	FASTERCNN	PlantVillage Dataset	Cotton	Model Customization	(X)	99.67%
[36]	2024	DCNN	Citrus leaf 5 Classes	Citrus	Transfer Learning with Image Augmentation	(X)	98%
[39]	2023	CNN on PaaS & DCNN	Tea Leaf	Tea	Tune Hyper-Parameter	(X)	100%
[41]	2024	DCNN	Potato leaf	Potato	Tune Hyper-Parameter Transfer Learning	(X)	99%
Our work	2024	AlexNet, GoogLeNet , InceptionV3 and VGG-19	Collected Dataset(Field) (600 Images)	Cotton	Transfer Learning Ensemble Learning	($\checkmark$)	98.4%

## Methodology

This section includes the collection of data, extracting features from the gathered data, and employing deep learning models for classification.

### Dataset collection

**Fig. 3:**
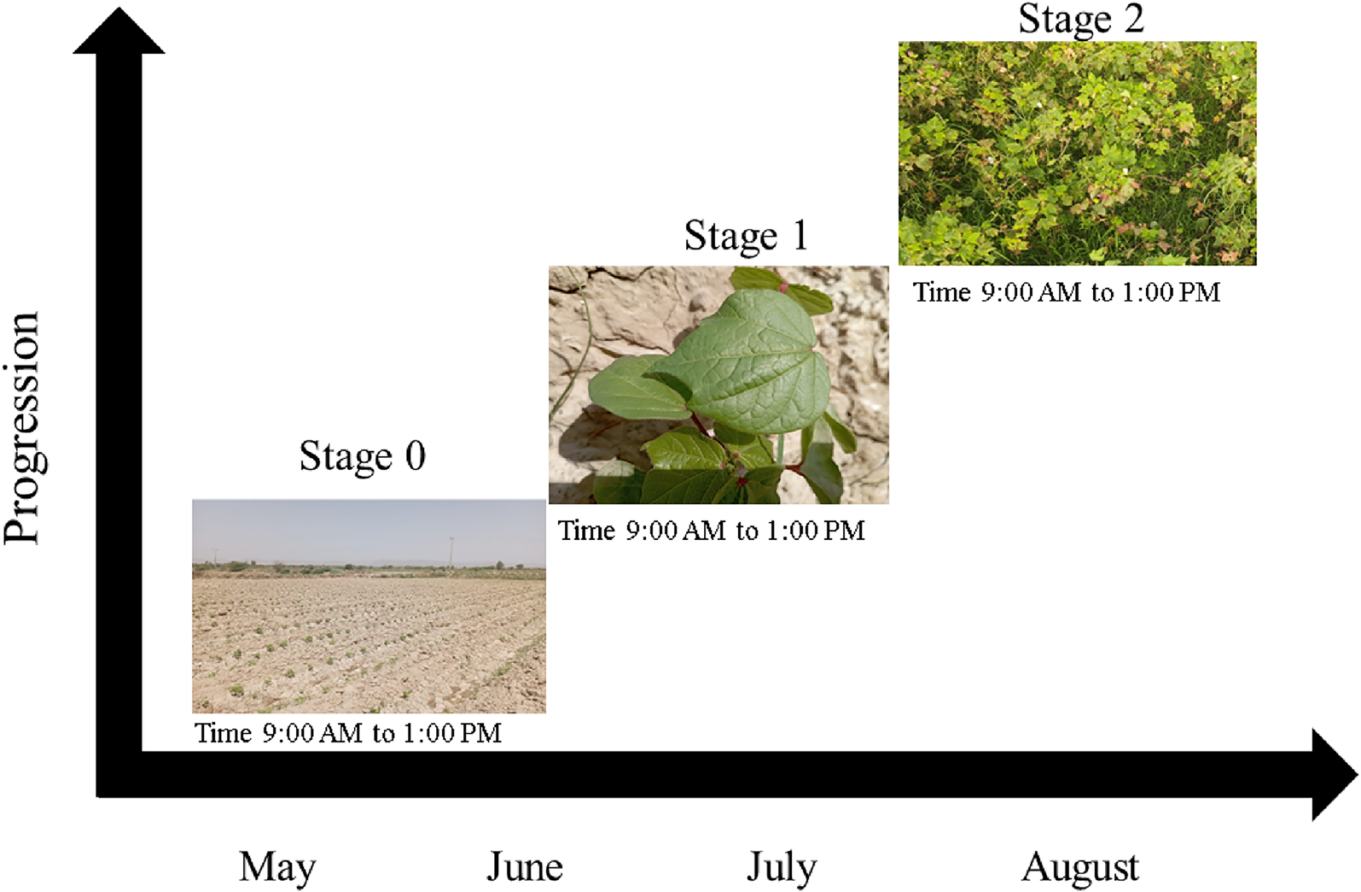
Data collection phases

**Fig. 4:**
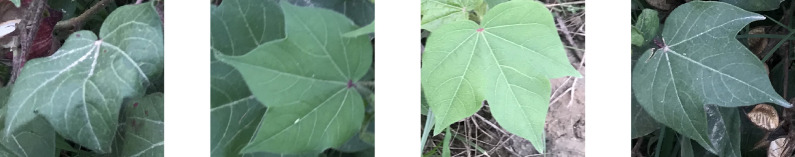
Healthy leaves sample images

**Fig. 5:**
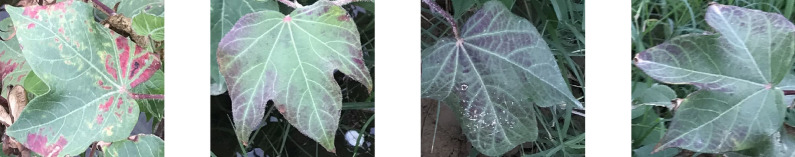
Unhealthy leaves sample images

The project is focused on the cotton fields situated in the Sindh region, encompassing areas within the observed region. Entire data-collection efforts were concentrated in the cotton fields of Sakran, a town in the Hub District of the province of Balochistan, Pakistan. Numerous visits were conducted to capture images for the dataset, facilitating comprehensive analysis. The dataset collection process was structured into three distinct phases as depicted in Fig. 3. The initial phase involved the period from May till June when the seed was sown into the soil, followed by the second phase from the last part of June and July, marked by the germination of the cotton plant. The final phase in August captured the stage when the cotton plant was fully matured and ready for cultivation. Figures 4 and  5 depict the sample of healthy and unhealthy plant. The rationale behind collecting the data in these phases was to conduct a comprehensive analysis of the plant’s condition at each stage, thereby enhancing the accuracy of classification. Additionally, the collected data serves a dual purpose, facilitating not only the immediate analysis but also serving as valuable training data for future model development, enabling the training of models from scratch.

### Image analysis and feature extraction

**Fig. 6:**
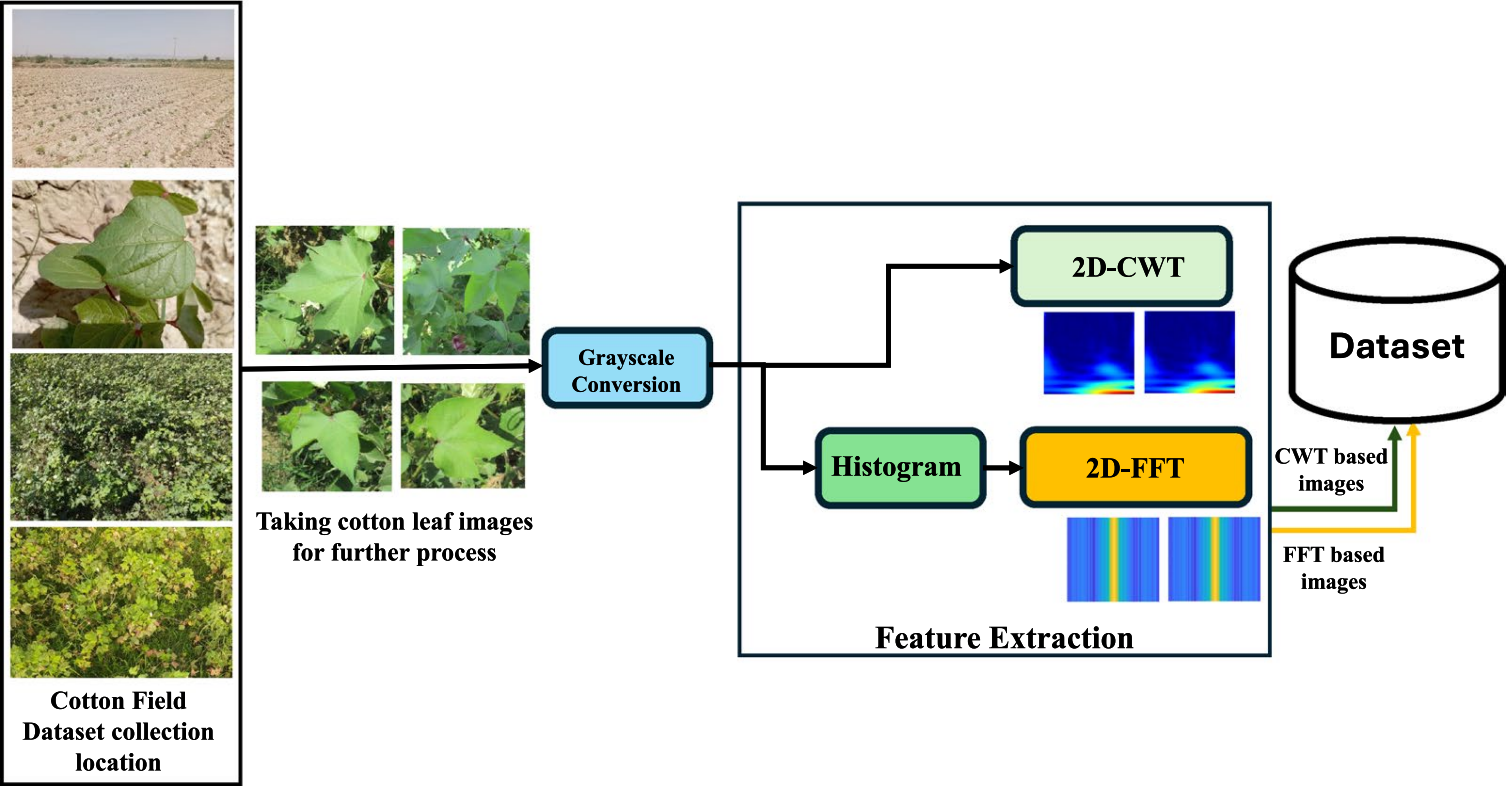
Feature extraction from the images using CWT and FFT

Features are extracted using CWT and FFT, using the steps depicted in Fig. 6.

Continuous wavelet transform

**Fig. 7:**
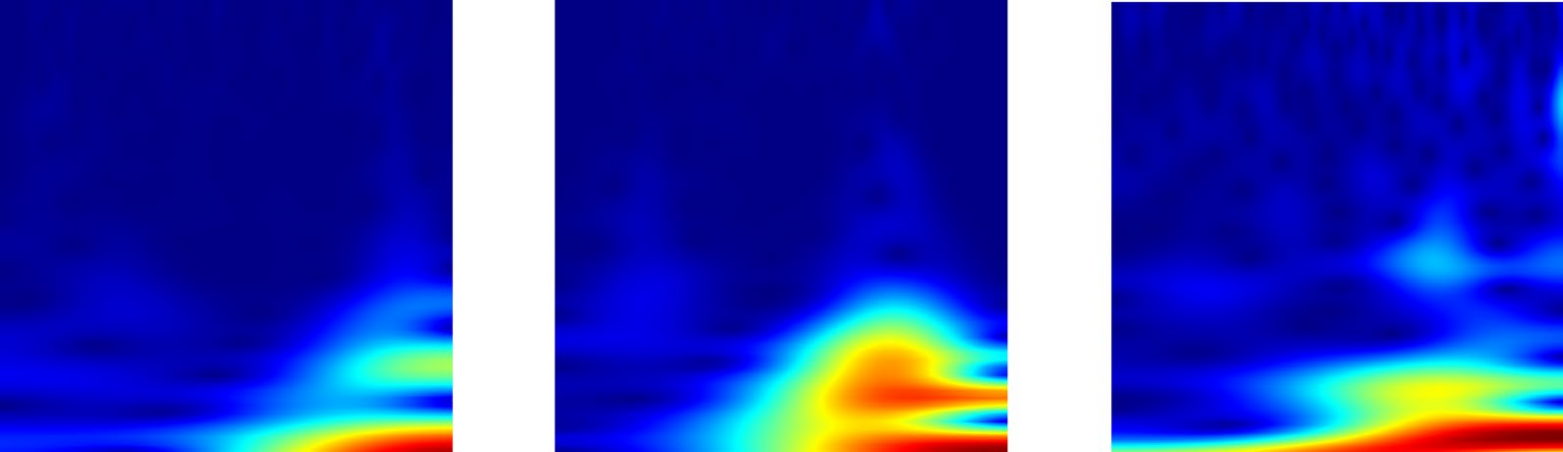
Extracted features using CWT from cotton plants

The 2D-CWT technique is utilized for the analysis and visualization of intricate patterns in cotton leaf images. Each original image is converted to grayscale to enhance the visibility of underlying patterns, facilitating a more concentrated examination of structural information. The visualization of scalograms offers a comprehensive illustration of frequency and scale components inherent in cotton leaf pictures as depicted in Fig. 7. The scalograms of diseased plants exhibit distinct temporal patterns, offering several advantages over raw red green blue (RGB) images. They prove valuable in capturing patterns and features at multiple scales. Their robustness in diverse environmental conditions, unaffected by lighting conditions, sets them apart. Additionally, they are computationally efficient due to dimensional reduction compared to RGB images. The scalogram can be displayed in two dimensions, with time on the horizontal axis and scale on the vertical axis. In this representation, the coefficient is color-coded using RGB values. Alternatively, the coefficient can be visualized in 3D contours, where the plot illustrates the energy associated with each coefficient. This has the potential to reveal previously unknown information about the characteristics of non-stationary processes. Scalograms find common application in diverse fields of vibration signal analysis, such as de-noising, structural analysis, ground motion analysis, fault diagnosis, damage detection, and more [72]. CWT scalograms are often more suitable due to their ability to capture both spatial and temporal variations in frequency and contribute to the broader field of image analysis and pattern recognition.

*Mathematical model*

In the CWT pathway, input images are transformed using $T_{CWT}(X)$, and the resulting wavelet representation $X_{CWT}$ is processed by a distinct deep learning model $M_{DL_{CWT}}$.

1 $$\begin{aligned}&\text {CWT Transformation:}&X_{CWT}&= T_{CWT}(I) \end{aligned}$$

2 $$\begin{aligned}&\text {Deep Learning Model Output:}&P_{DL_{CWT}}(C | X_{CWT})&= M_{DL_{CWT}}(X_{CWT}) \end{aligned}$$

Fast Fourier transform

**Fig. 8:**
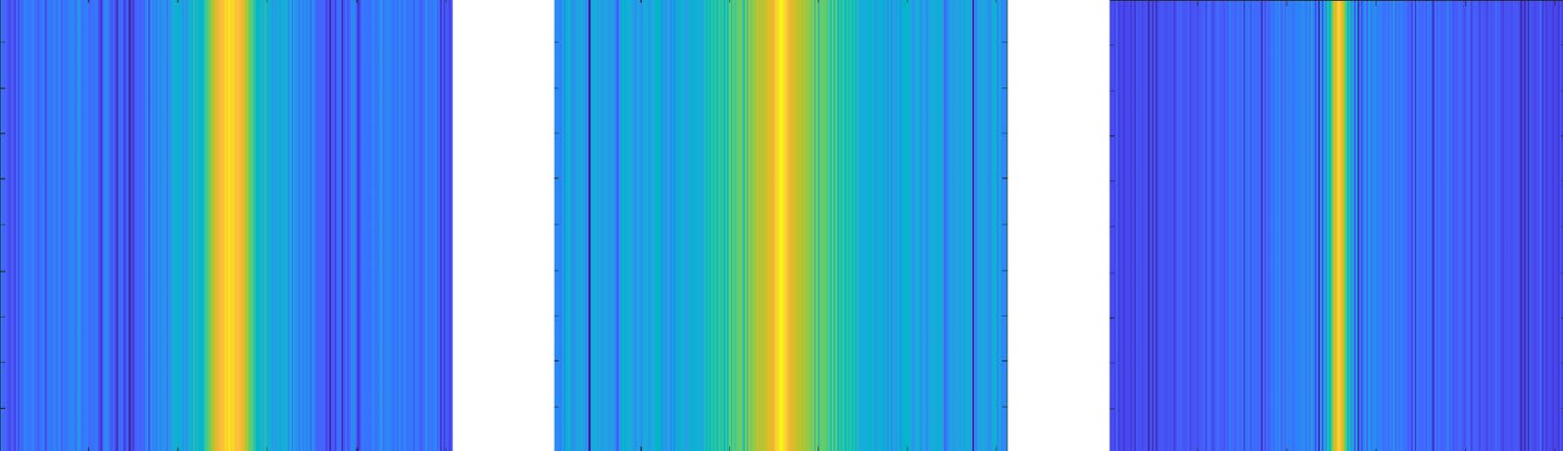
Extracted features using FFT from cotton plants

Color histograms were generated as features from individual leaf images. Subsequently, the 2D FFT was applied to each row of the feature matrix, allowing for the visualization of the FFT magnitude as shown in Fig. 8. This approach aids in the examination of frequency components within the images, offering valuable insights into their structural attributes. The resulting images were saved in a designated directory for classification purposes.

*Mathematical model*

In the FFT pathway, input images are transformed using $T_{FFT}(X)$ and the resulting frequency-domain representation $X_{FFT}$ is fed into a dedicated deep learning model $M_{DL_{FFT}}$.

3 $$\begin{aligned}&\text {FFT Transformation:}&X_{FFT}&= T_{FFT}(X) \end{aligned}$$

4 $$\begin{aligned}&\text {Deep Learning Model Output:}&P_{DL_{FFT}}(C | X_{FFT})&= M_{DL_{FFT}}(X_{FFT}) \end{aligned}$$

### Deep learning models

In the pursuit to classify healthy and unhealthy cotton leaves, multiple pre-trained deep learning models are trained using the collected dataset. The models utilized in this process include AlexNet, GoogLeNet, VGG-19, and InceptionV3. The training process involved using the adaptive moment estimation (Adam) optimizer function, maintaining a batch size of 32, setting the initial learning rate to 5e-5, and running for a total of 100 epochs as shown in Table 3.

**Table 3:** Hyperparameters of deep learning models

Models	No. of epochs	Optimizer	Batch size	Initial learning rate
AlexNet	100	Adam	32	5.00e-05
GoogLeNet	100	Adam	32	5.00e-05
InceptionV3	100	Adam	32	5.00e-05
VGG-19	100	Adam	32	5.00e-05

#### AlexNet

In 2012 [73], Alex Krizhevesky et al. presented a deeper and larger CNN model than LeNet, winning the ImageNet Large Scale Visual Detection Challenge (ILSVRC), which is the most prestigious ImageNet challenge for visual object detection [74]. In terms of recognition accuracy, it surpassed all traditional machine learning and computer vision techniques. The architecture of AlexNet is distinctive due to its innovative design, comprising five convolutional layers followed by three fully connected layers. The convolutional layers employ filter sizes ranging from 11x11 to 3x3, with a stride of 4 in the first layer and 1 in subsequent layers. This configuration allows the network to effectively capture spatial hierarchies of features. Notably, using ReLU activation functions speeds up model training by fast convergence. The introduction of local response normalisation (LRN) in the architecture improves the network’s ability for generalisation.

#### GoogLeNet

GoogLeNet, commonly known as Inception v1, has a distinct and complex image classification architecture. Google introduced it in 2014, and its defining feature is the revolutionary “Inception” module [75]. These modules use parallel convolutions with filter sizes of 1x1, 3x3, and 5x5, as well as max pooling, to collect information at several spatial scales. This architectural choice improves the model’s recognition of complicated patterns in photos. 1x1 convolutions are employed strategically to reduce dimensionality to overcome computational issues associated with large filter sizes. During training, the network includes auxiliary classifiers at intermediate layers to assist in gradient flow and mitigate potential training challenges. The auxiliary classifier is comprised of an average pooling layer (5$\times$5 filter, stride 3), followed by a 1$\times$1 convolution (128 filters, ReLU activation), a fully connected layer (1025 outputs, ReLU activation), and dropout regularization (dropout ratio of 0.7). The architecture is completed with a softmax classifier that outputs 1000 classes, aligning with the main softmax classifier to address the vanishing gradient problem. The overall design of GoogLeNet aims to strike a balance between model complexity and computational efficiency.

#### VGG-19

Simonyan and Zisserman introduced VGG19 [76], an impressive Convolutional Neural Network (CNN) with 19 layers, comprising 16 convolutional layers and 3 fully connected layers. This network is specifically designed for classifying photos into 1000 categories. VGG19 was trained on the ImageNet database, containing a million pictures classified into 1000 categories. This powerful image classification technique is recognized for its utilization of a substantial number of 3x3 filters in each convolutional layer. This means that sixteen convolutional layers were used for feature extraction, followed by three layers for classification. The feature extraction layers are divided into five groups, each followed by a max-pooling layer. This model processes 224-by-224 pictures and outputs the object’s label. The classification in the study is done with a pre-trained VGG19 model.

#### InceptionV3

InceptionV3, a profound CNN architecture designed for the classification of images and identification of objects, is an advancement of the original Inception model [77]. Google introduced it in 2016 as part of its GoogLeNet family. By further enhancing the “Inception” module design, InceptionV3 builds on the success of its predecessors. A highly sophisticated network composed of numerous stacked Inception modules, each combining concurrent convolutions of varying filter sizes (1x1, 3x3, and 5x5) and pooling operations, is featured in the architecture. Batch normalization is utilized to expedite training by normalizing the inputs of each layer. The computational expense of Inception is significantly less than that of VGGNet or its more advanced counterparts [78]. As a result, Inception networks can now be used in big-data applications.

#### Ensemble model

**Fig. 9:**
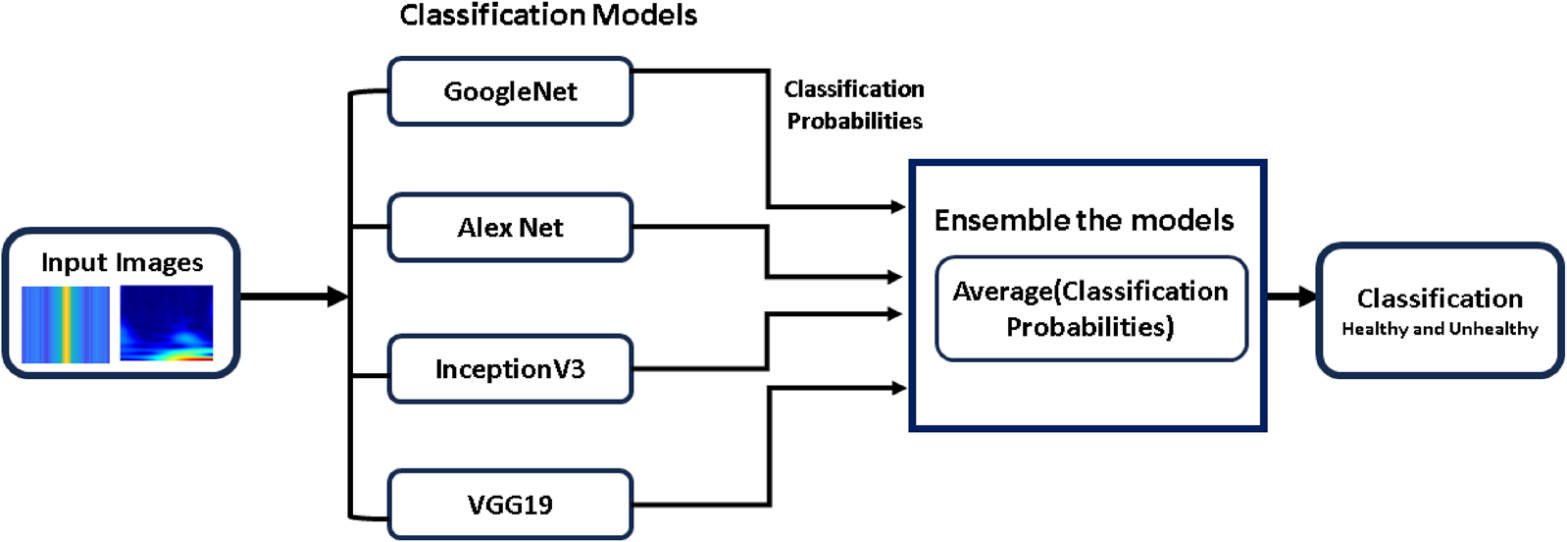
Ensemble learning approach to enhance detection score

Ensemble learning, as discussed in reference [79], is a robust technique in machine learning that involves combining the predictions of multiple models to enhance overall performance. This is achieved by leveraging diverse models, each with its unique strengths and weaknesses, ensemble methods can produce more robust and accurate predictions compared to individual models [80]. One popular ensemble methodology is averaging [81], where the classification probabilities of each model are aggregated to compute a mean probability. This approach helps mitigate the impact of outliers and model biases, resulting in a more balanced and reliable decision-making process. In our study, we implemented an averaging ensemble method using four pre-trained models-GoogLeNet, AlexNet, InceptionV3, and VGG19-for classification tasks as shown in Fig. 9. The utilization of these distinct architectures, combined with the averaging approach, led to superior model performance, surpassing the classification capabilities of any individually trained model.

**Algorithm 1:**
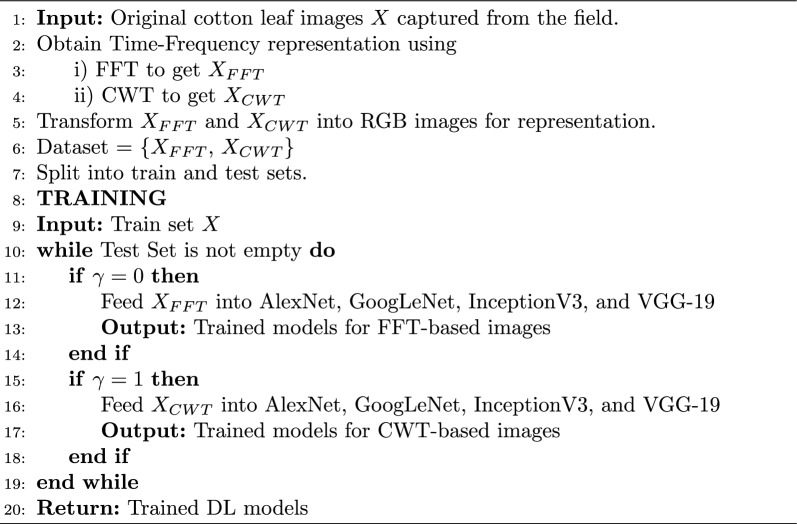
Deep learning models training

**Algorithm 2:**
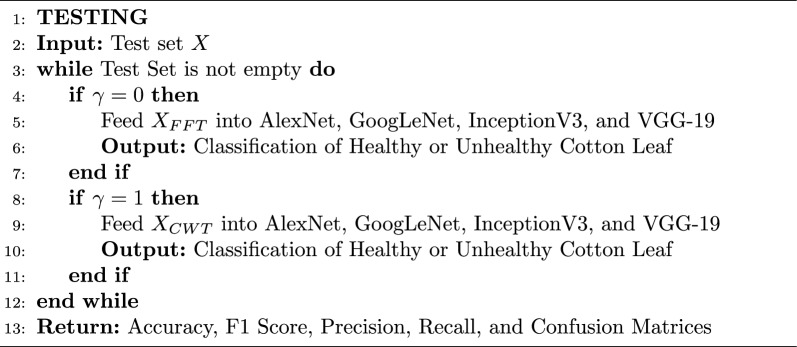
Deep learning models testing

## Experiment, setup, and results

**Fig. 10:**
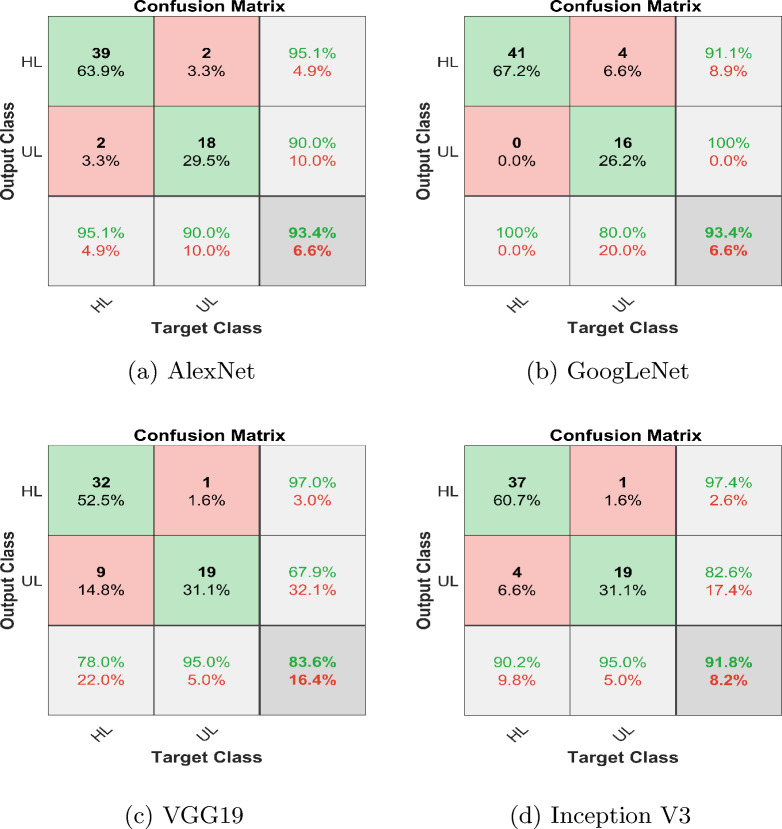
Confusion matrix wavelet based classification

**Fig. 11:**
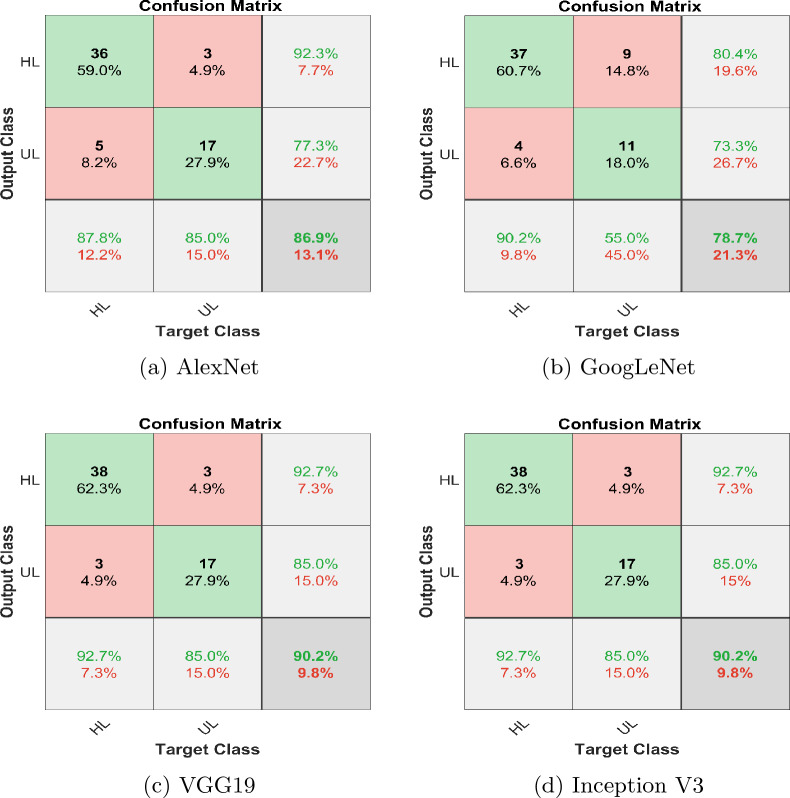
Confusion matrix FFT based classification

The implementation consists of the proposed method consisting of training and testing phases and for that purpose, MATLAB is selected as a simulation platform. Features extraction using CWT and FFT was done on the collected cotton dataset. The dataset is divided into a 70:30 split ratio for training and test sets. As mentioned above we use GoogleNet, AlexNet, VGG-16, and Inception-V3 as a deep learning model for classification tasks. Subsequently, the output layer of the DL models is configured to classify two classes instead of 1000 which is the default value. The results were evaluated based on accuracies and losses of train and test sets. Furthermore, precision, F1-score, and recall were calculated to reaffirm the obtained results. The overall implementation of training and testing is shown Algorithms 1 and  2. The corresponding statistical values are also presented in Table 4 for CWT and Table 5 for FFT. To assess the efficiency of the trained deep learning models, confusion matrices were generated and visualized in Fig. 10 for CWT and Fig. 11 for FFT. These visual representations provide insights into the model’s performance in distinguishing between healthy and unhealthy cotton leaf images. Moreover, to ensure the robustness of of our trained models and minimise the biases of the distribution of dataset K-Fold cross validation is applied, where the value of K is set to 5, the detailed analysis is shown in Tables 6 and  7.

**Fig. 12:**
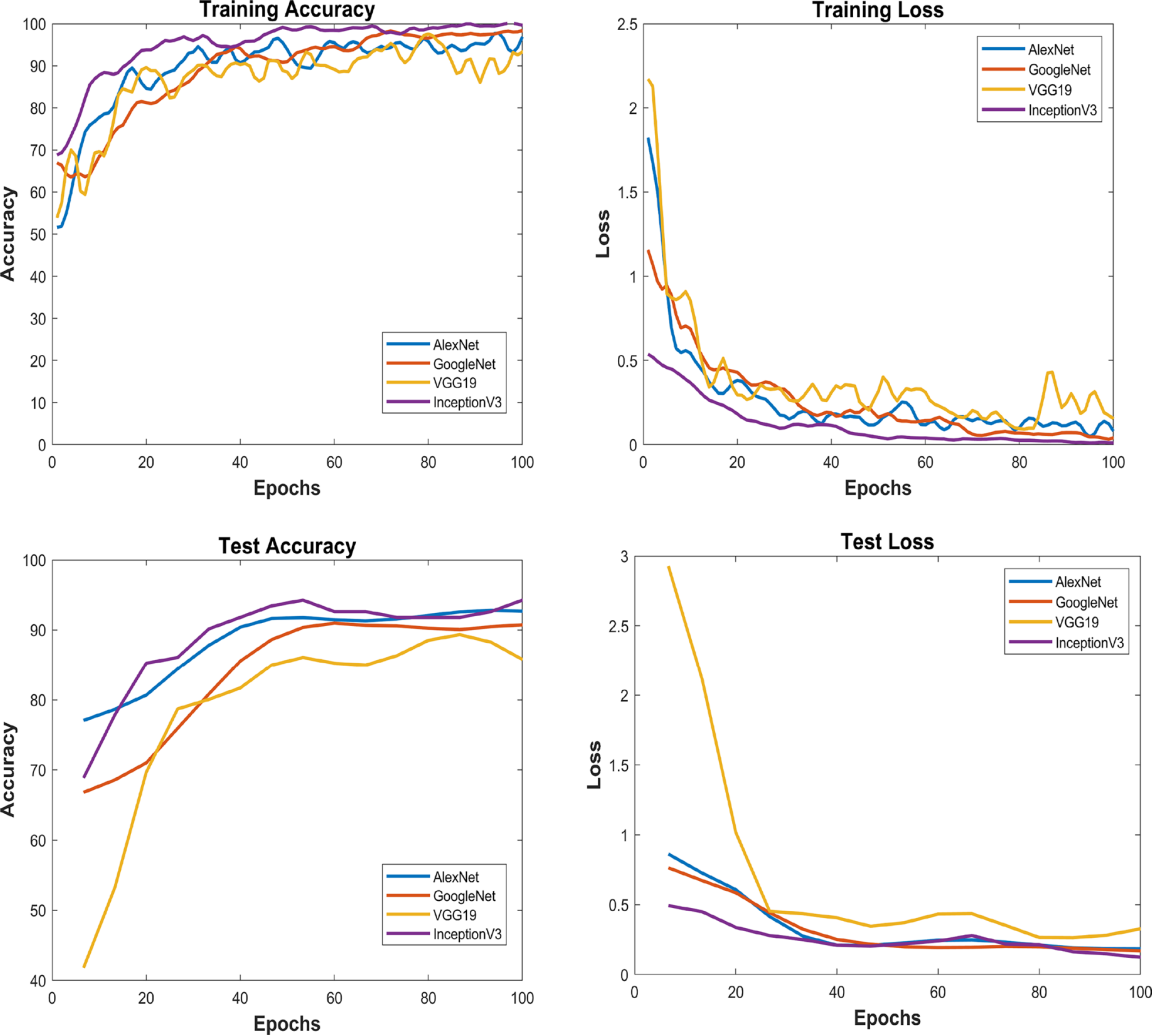
Wavelet images classification results- Left upper corner plot shows training accuracy of all dl models, right upper corner plot shows training loss, left lower corner plot depicts test accuracy of dl models, right lower corner plot demonstrate test loss

**Fig. 13:**
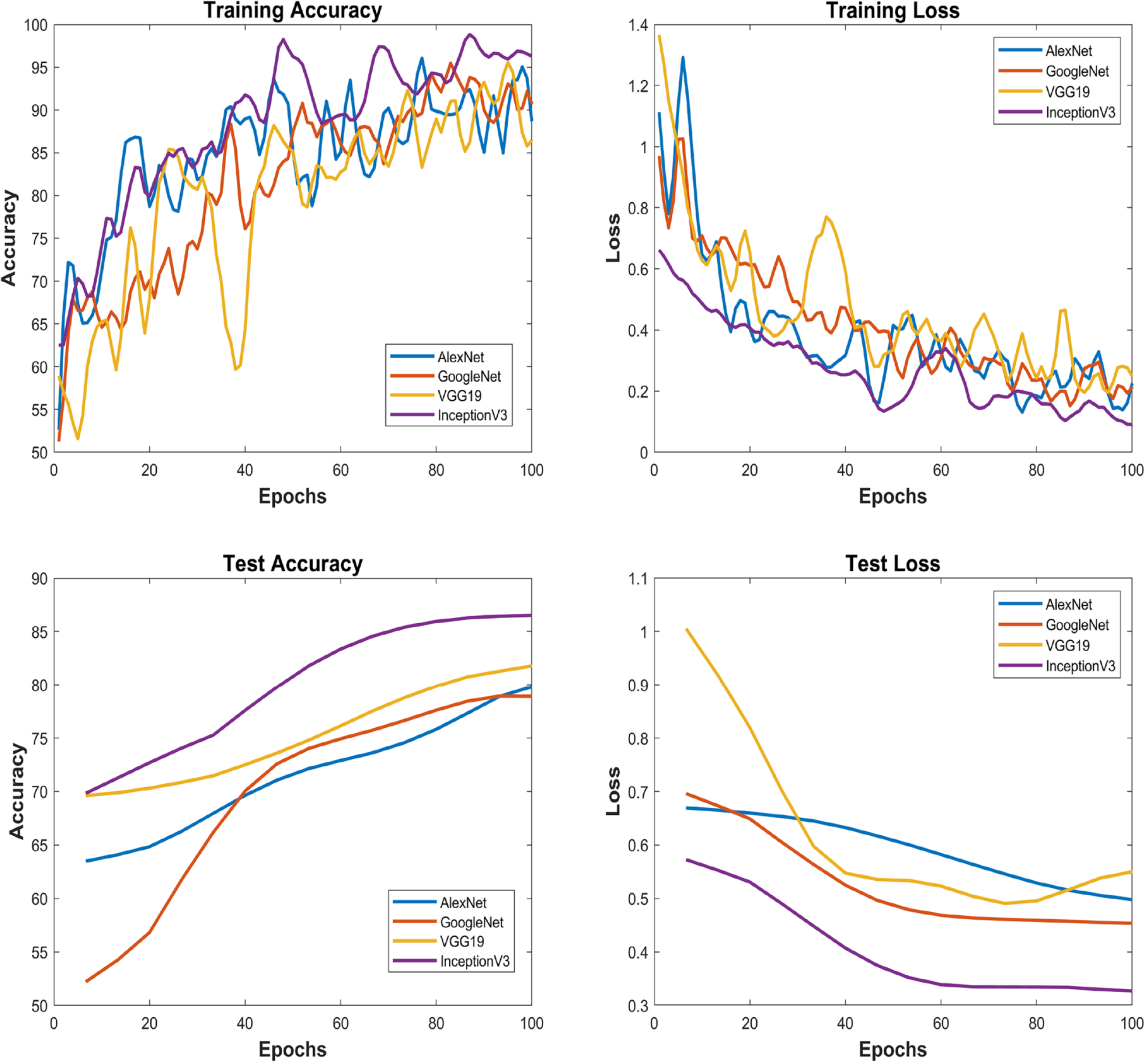
FFT images classification results-left upper corner plot shows training accuracy of all dl models, right upper corner plot shows training loss, left lower corner plot depicts test accuracy of dl models, right lower corner plot demonstrate test loss

**Table 4:** Evaluation parameters for wavelet images based classification

Features extracted using CWT					
Models	Training accuracy (%)	Test accuracy (%)	Precision	Recall	F1-score
GoogLeNet	99.81	93.4	0.911	1.000	0.953
AlexNet	96.86	93.4	0.951	0.951	0.951
VGG-19	95.80	83.6	0.970	0.780	0.865
InceptionV3	99.92	91.8	0.974	0.902	0.937
Ensemble	99.92	98.40	0.952	1.000	0.976

**Table 5:** Evaluation parameters for FFT images based classification

Features extracted using FFT					
Models	Training accuracy (%)	Test accuracy (%)	Precision	Recall	F1-score
GoogLeNet	96.87	78.7	0.804	0.902	0.851
AlexNet	93.75	86.9	0.923	0.878	0.900
VGG-19	90.62	90.2	0.927	0.927	0.927
InceptionV3	98.96	90.2	0.927	0.927	0.927
Ensemble	98.96	95.10	0.947	0.900	0.923

**Fig. 14:**
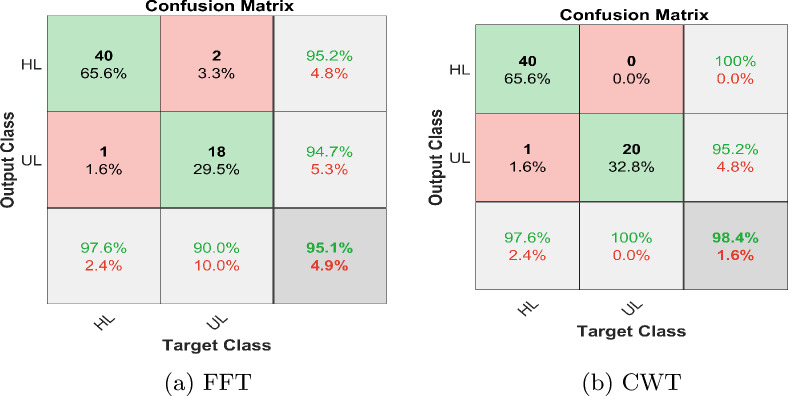
Confusion matrix; ensemble learning

**Table 6:** Performance analysis using K-fold on CWT

Features extracted using CWT						
Models	K-fold 1 (%)	K-fold 2 (%)	K-fold 3 (%)	K-fold 4 (%)	K-fold 5 (%)	Average (%)
GoogleNet	91.67	93.44	88.52	90.00	95.08	91.74
AlexNet	95.00	95.00	92.42	83.61	91.89	91.58
VGG-19	93.33	90.16	95.00	91.80	89.80	92.02
InceptionV3	98.33	93.44	91.80	90.00	96.72	94.06

**Table 7:** Performance analysis using K-fold on FFT

Features extracted using FFT						
Models	K-fold 1 (%)	K-fold 2 (%)	K-fold 3 (%)	K-fold 4 (%)	K-fold 5 (%)	Average (%)
GoogleNet	90.16	86.89	91.67	90.16	90.00	89.78
AlexNet	93.44	90.16	93.33	86.67	86.89	90.10
VGG-19	88.33	88.52	95.00	88.52	95.08	91.09
InceptionV3	95.00	95.08	95.08	93.44	90.00	93.72

**Table 8:** Computational statistic for the applied models $CWT-Scalograms$

CWT- scalograms				
Models	Processing time (training) in S	Processing time (prediction) in S	Memory consumption (training) in KBs	Memory consumption (prediction) in KBs
AlexNet	700.512	0.0692	17316.21	0.8907
GoogLeNet	1521.72	0.2237	5260.25	0.8907
InceptionV3	6713.94	0.5380	8123.81	0.8907
VGG-19	15061.54	0.3070	17284.51	0.8907

**Table 9:** Computational statistic for the applied models $FFTs$

FFT				
Model	Processing time (training) in S	Processing time (prediction) in S	Memory consumption (training) in KBs	Memory consumption (prediction) in KBs
AlexNet	750.595	0.066	17316.22	0.8904
GoogLeNet	1891.98	0.183	5308.56	0.8904
InceptionV3	6713.94	0.538	8123.81	0.8904
VGG-19	1224.61	0.298	17284.52	0.8904

**Fig. 15:**
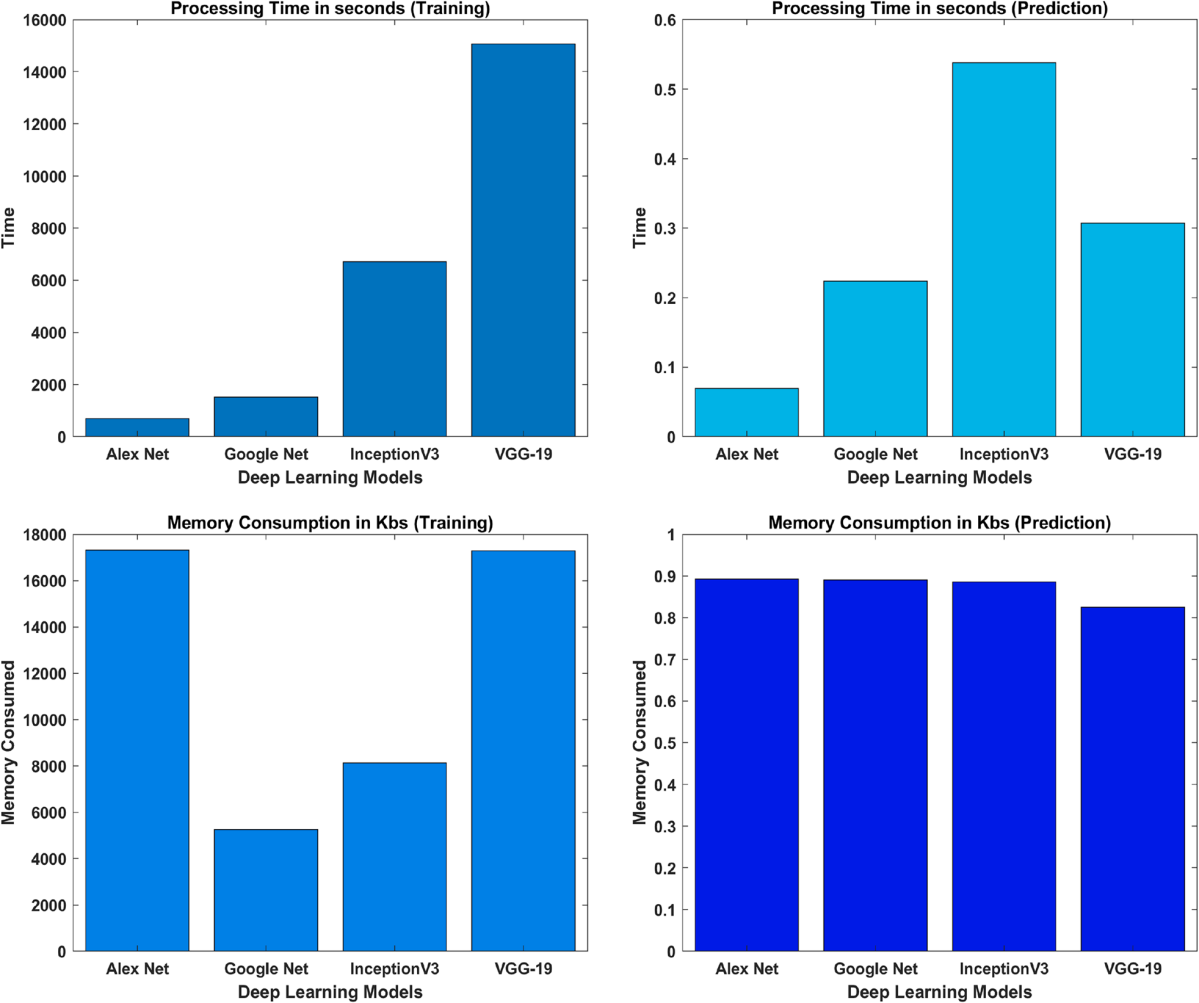
Performance analysis of models trained on CWT scalograms

**Fig. 16:**
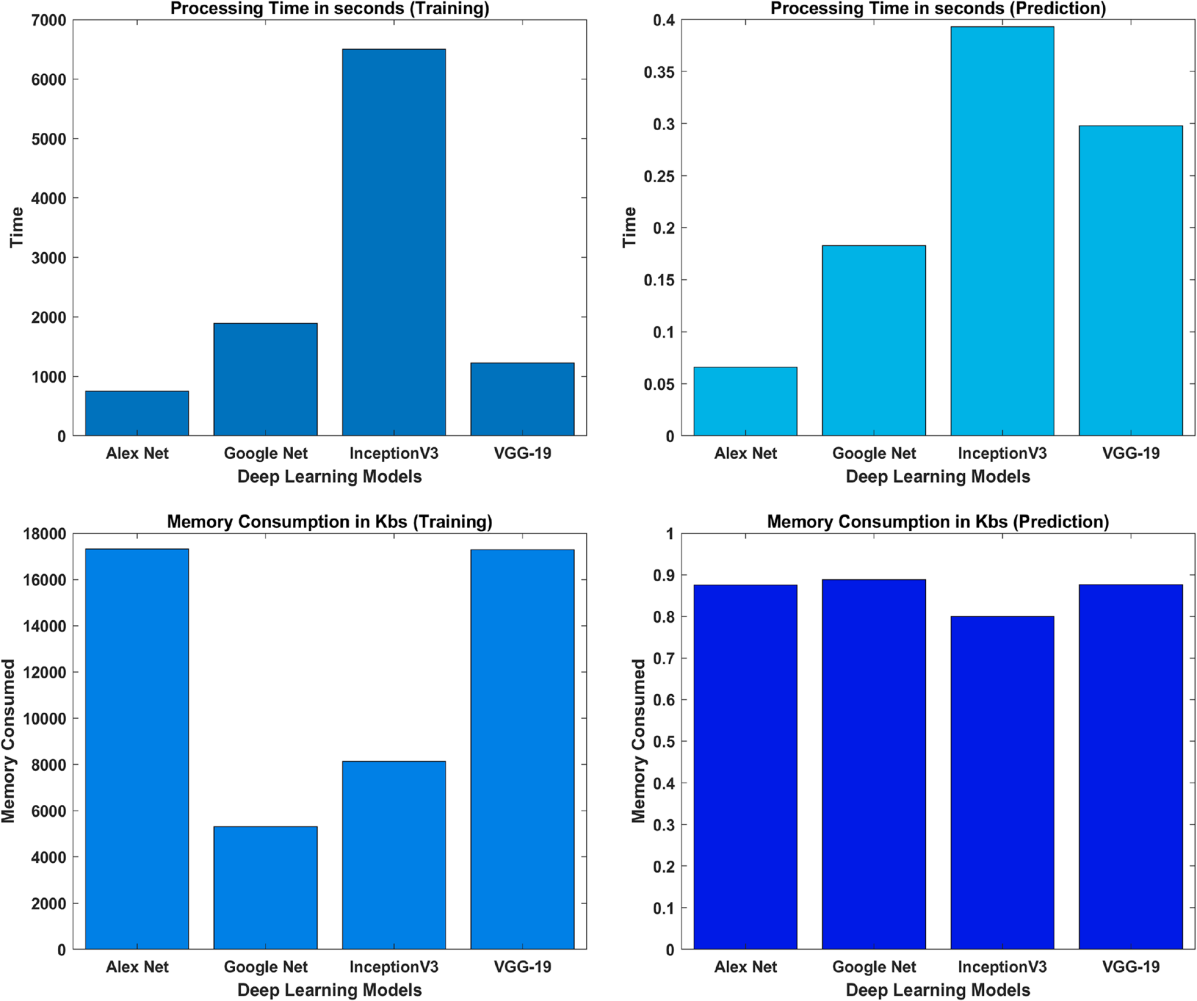
Performance analysis of models trained on FFT images

**Fig. 17:**
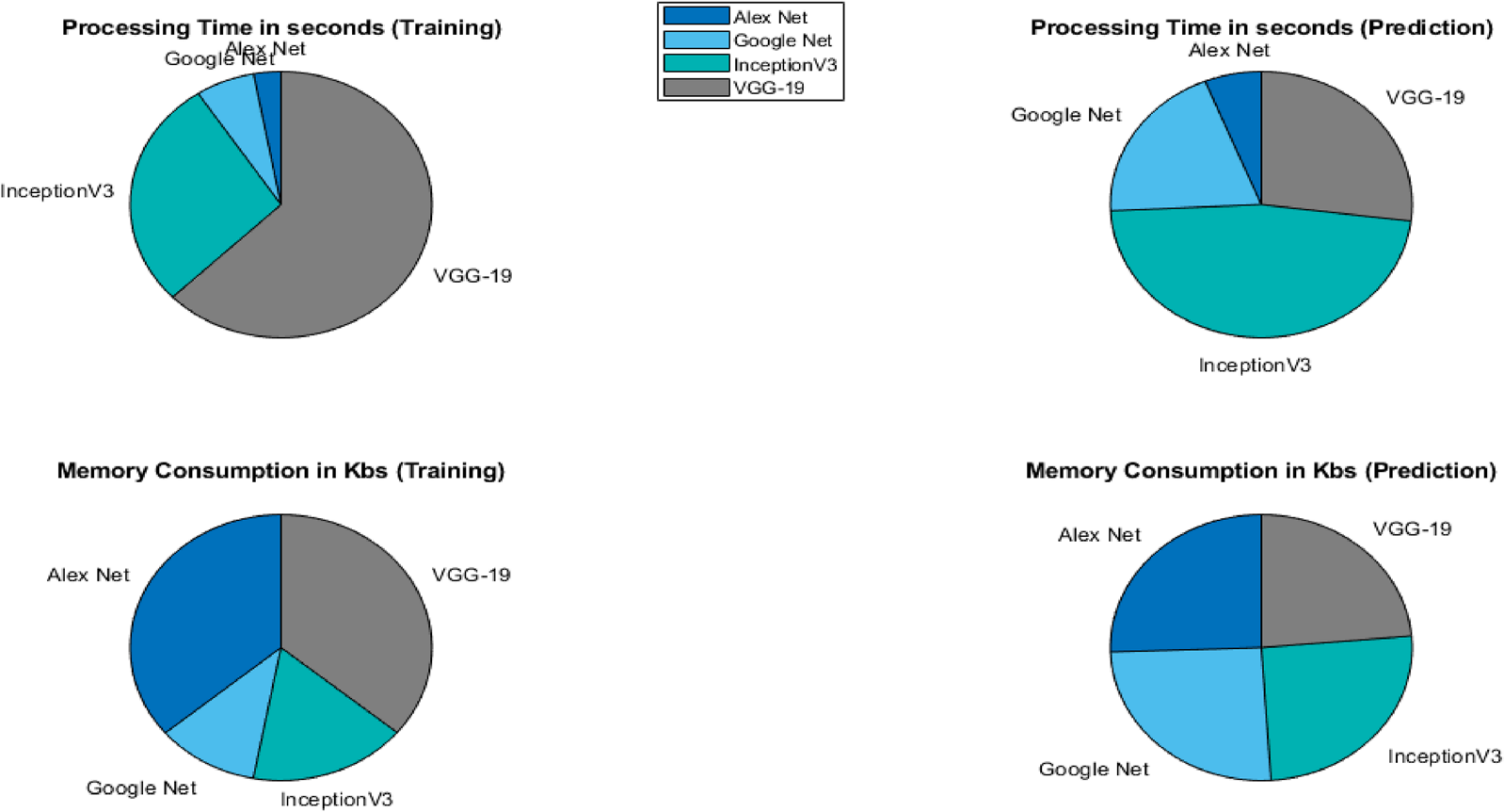
Performance analysis (CWT)

**Fig. 18:**
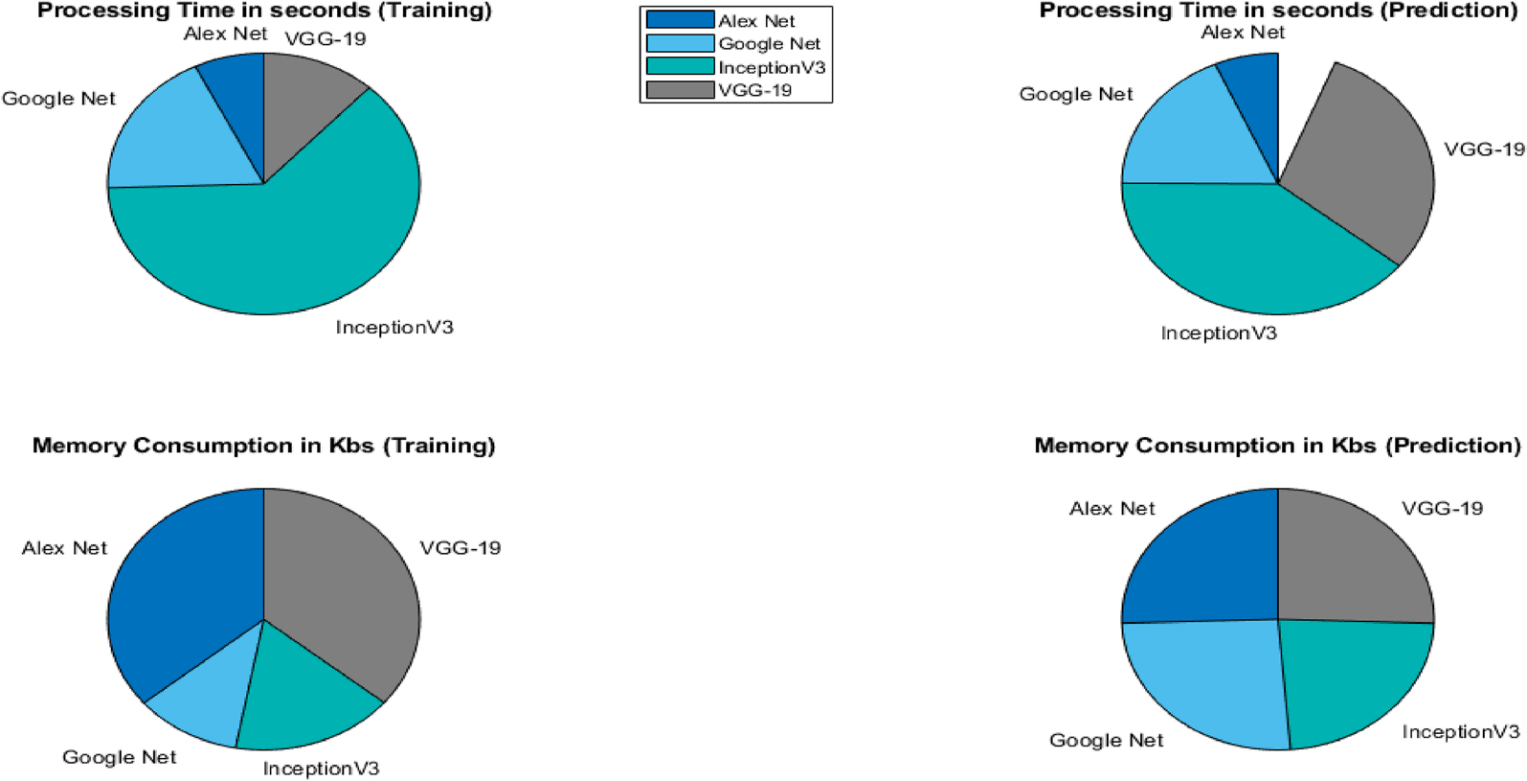
Performance analysis (FFT)

In this comprehensive analysis employing both CWT and FFT for image analysis and feature extraction, deep learning models-GoogLeNet, AlexNet, VGG-19, and InceptionV3-were evaluated. Utilizing CWT-based images, GoogLeNet exhibited exceptional precision, recall, and F1-Score, achieving a 99.81% training accuracy and 93.4% testing accuracy. AlexNet demonstrated balanced performance with 96.86% training accuracy and 93.4% testing accuracy. VGG-19 and InceptionV3, while achieving high training accuracies, showed variations in precision, recall, and F1-Score. Figure 12 depicts training accuracy and loss plots, and test accuracy and loss plots. Transitioning to FFT-based images, InceptionV3, with a training accuracy of 98.96% and testing accuracy of 90.2%, demonstrates solid generalization capabilities. VGG-19, despite a slightly lower training accuracy of 90.62%, achieves a good testing accuracy of 90.2%. Figure 13 depicts training accuracy and loss plots, and test accuracy and loss plots. Consequently, comparing the two potential techniques, the results suggest that the CWT-based approach yielded higher precision, recall, and overall F1-Score, showcasing its superior performance in identifying unhealthy cotton leaf images.

After training the models individually and consolidating the results, a deep ensemble model employing the averaging method was utilized. This enables the ensemble model to utilize prediction probabilities from each model and carry out the classification. Ensemble learning is a powerful method that fuses the classification scores of individual classifiers to enhance the overall classification score. Based on our implementation of deep learning models to detect plant conditions on individual basis, we chose to ensemble the best four models in the ensemble framework to enhance the detection capability of our proposed framework and it is evident from Tables 4 and  5 that overall performance has been improved due to the ensemble method. The outcomes demonstrate that ensembling the models enhances classification test accuracies, 98.40% and 95.10% using CWT-scalograms and FFTs, respectively as shown in Fig. 14. Furthermore, we calculated the computational statistics of implemented models in terms of processing time and memory consumption for both training and testing. We did it for both CWT and FFT, these values are listed in Tables 8 and  9 respectively. The graphical representation of the same is shown in Figs. 15 and  16. It shows that AlexNet is the fastest model for both training and prediction. Additionally, GoogLeNet exhibits the most efficient memory consumption among the models. The pie-charts in Figs. 17 and  18 present a better graphical representation of the performance of the trained models.

## Conclusion and future work

In this paper, we endeavor to advance the accuracy and efficacy of disease detection in plants specifically within the domain of cotton using cutting-edge deep-learning models and visualization techniques. Our approach transforms the real-time images captured from the field using CWT and FFT before they are fed into the DL models. The process of collecting the dataset on the locally observed field was methodically structured into three specific phases. The purpose behind this approach was to conduct a thorough analysis of the plant’s condition at each stage, ultimately improving classification precision. The results obtained from the evaluation show that CWT-transformed images outperform FFT-transformed images in the classification of healthy and unhealthy cotton plants. Employed DL models GoogLeNet, AlexNet, VGG-19, and InceptionV3 all attained high recognition accuracy and better F1-score, recall, and precision when trained using CWT scalograms. GoogLeNet was the superior model with a test accuracy of 93.4%. InceptionV3 had the greatest FFT-trained recognition accuracy of 98.96% and test accuracy of 90.2%. Furthermore, after training and testing each model separately, we applied an ensemble method based on their classification probabilities. The ensemble method averaged the weak individual learner models to create a stronger collective model. The ensemble model outperformed individual models with an exceptional test accuracy of 98.4% after including CWTs. Significant disease control using pre-trained models which use CWT scalograms can mitigate significant plant damage, leading to higher yields and improved harvest quality. This translates into increased agricultural earnings and a more reliable cotton supply chain. However, several obstacles remain, such as the availability of computational resources and the costs involved with adoption. Current research in the field seeks to address these limits. Overall, the findings demonstrate the effectiveness of using cutting-edge technology to improve plant disease diagnosis, opening up new paths for future study and practical applications in agricultural management and crop health monitoring. Future research efforts could include adding unmanned aerial vehicles (UAVs) for data collection and implementing lightweight models such as MobileNet for real-time classification. This not only speeds up the data processing pipeline but also enables faster and more informed farm decisions. Farmers can gain considerably from such an approach since it allows them to cover larger land areas in less time, facilitating efficient crop management practices.

## Data Availability

The datasets generated and/or analysed during the current study are not publicly available but are available from the corresponding author upon reasonable request.

## References

[1] Worldometer: largest countries in the world by area 2023;2023. https://www.worldometers.info/geography/largest-countries-in-the-world/.

[2] Jung J, Maeda M, Chang A, Bhandari M, Ashapure A, Landivar-Bowles J (2021). The potential of remote sensing and artificial intelligence as tools to improve the resilience of agriculture production systems. Curr Opin Biotechnol.

[3] Toda Y, Okura F. How convolutional neural networks diagnose plant dis-542 ease. Plant Phenomics 2019. 10.34133/2019/9237136 10.34133/2019/9237136 PMC7706313 33313540

[4] Khan MA, Wahid A, Ahmad M, Tahir MT, Ahmed M, Ahmad S. Hasanuzzaman M. World Cotton Production and Consumption: An Overview, Springer (2020). pp. 1–7. 10.1007/978-981-15-1472-21

[5] Azumah SB, Donkoh SA, Awuni JA (2019). Correcting for sample selection in stochastic frontier analysis: insights from rice farmers in northern ghana. Agric Food Econ..

[6] Abbas S, Waheed A (2017). Trade competitiveness of pakistan: evidence from the revealed comparative advantage approach. Compet Rev Int Bus J..

[7] Razzaq A, Zafar MM, Ali A, Hafeez A, Batool W, Shi Y, Gong W, Yuan Y (2021). Cotton germplasm improvement and progress in pakistan. J Cotton Res..

[8] Shuli F, Jarwar AH, Wang X, Wang L, Ma Q (2018). Overview of the cotton in pakistan and its future prospects. Pak J Agric Res..

[9] Shahbandeh M. Cotton statistics and facts Statista. Statista. 2023. https://www.statista.com/topics/3934/cotton-statistics-facts/.

[10] Pakistan Bureau of Statistics. Pakistan Bureau of Statistics. https://www.pbs.gov.pk/agriculture-statistics-tables. Accessed 20 Dec 2023.

[11] Ip RH, Ang L-M, Seng KP, Broster J, Pratley J (2018). Big data and machine learning for crop protection. Comput Electron Agric.

[12] Chen J, Chen J, Zhang D, Sun Y, Nanehkaran YA (2020). Using deep transfer learning for image-based plant disease identification. Comput Electron Agric.

[13] Lu J, Tan L, Jiang H (2021). Review on convolutional neural network (cnn) applied to plant leaf disease classification. Agriculture.

[14] Verma S, Chug A, Singh AP. Prediction models for identification and diagnosis of tomato plant diseases. In: 2018 International Conference on Advances in Computing, Communications and Informatics (ICACCI), 2018;1557–1563. 10.1109/ICACCI.2018.8554842

[15] Gebbers R, Adamchuk VI (2010). Precision agriculture and food security. Science.

[16] Deshapande AS, Giraddi SG, Karibasappa K, Desai SD. Fungal disease detection in maize leaves using haar wavelet features. In: Information and Communication Technology for Intelligent Systems: Proceedings of ICTIS 2018, Volume 1, pp. 2019;275–286 Springer.

[17] Dhaygude SB, Kumbhar NP (2013). Agricultural plant leaf disease detection using image processing. Int J Adv Res Electric Electron Instrum Eng.

[18] Hall D, McCool C, Dayoub F, Sunderhauf N, Upcroft B. Evaluation of features for leaf classification in challenging conditions. In: 2015 IEEE winter conference on applications of computer vision. IEEE;2015. pp. 797–804.

[19] Li L, Zhang S, Wang B (2021). Plant disease detection and classification by deep learning-a review. IEEE Access.

[20] Camargo A, Smith JS (2009). Image pattern classification for the identification of disease causing agents in plants. Comput Electron Agric.

[21] Miao M, Hu W, Yin H, Zhang K. Spatial-frequency feature learning and classification of motor imagery eeg based on deep con- volution neural network. Computational and Mathematical Methods in Medicine. 2020;(1):1981728. 10.1155/2020/1981728 10.1155/2020/1981728 PMC7387988 32765639

[22] Rhif M, Ben Abbes A, Farah IR, Martínez B, Sang Y (2019). Wavelet transform application for/in non-stationary time-series analysis: A review. Appl Sci.

[23] Moravej Z, Mortazavi SH, Shahrtash SM (2015). Dt-cwt based event feature extraction for high impedance faults detection in distribution system. Int Trans Electric Energy Syst.

[24] Min D, Jiuwen Z, Yide M (2015). Image denoising via bivariate shrinkage function based on a new structure of dual contourlet transform. Signal Process.

[25] King SL, Bennett KP, List S (2000). Modeling noncatastrophic individual tree mortality using logistic regression, neural networks, and support vector methods. Comput Electron Agric.

[26] Chai A, Li B, Shi Y, Cen Z, Huang H, Liu J (2010). Recognition of tomato foliage disease based on computer vision technology. Acta Horticult Sin.

[27] Yao C, Zhang Y, Liu H (2017). Application of convolutional neural network in classification of high resolution agricultural remote sensing images. Int Arch Photogramm Remote Sens Spat Inf Sci.

[28] Kessentini Y, Besbes MD, Ammar S, Chabbouh A (2019). A two-stage deep neural network for multi-norm license plate detection and recognition. Expert Syst Appl.

[29] Sladojevic S, Arsenovic M, Anderla A, Culibrk D, Stefanovic D. Deep neural networks based recognition of plant diseases by leaf image classification. Computational Intelligence and Neuro- science. 2016;(1):3289801. 10.1155/2016/3289801 10.1155/2016/3289801 PMC4934169 27418923

[30] Mohanty SP, Hughes DP, Salathé M (2016). Using deep learning for image-based plant disease detection. Front Plant Sci.

[31] Bhagat SK, Tiyasha T, Tung TM, Mostafa RR, Yaseen ZM (2020). Manganese (mn) removal prediction using extreme gradient model. Ecotoxicol Environ Saf.

[32] Abd Elaziz M, Mabrouk A, Dahou A, Chelloug SA. Medical image classifi- cation utilizing ensemble learning and levy flight-based honey badger algorithm on 6g-enabled internet of things. Computational Intelligence and Neuroscience 2022;(1):5830766 28 10.1155/2022/5830766 PMC9168094 35676950

[33] Zhang S, Huang W, Zhang C. Three-channel convolutional neural networks for vegetable leaf disease recognition. Cogn Syst Res. 2018;53. 10.1016/j.cogsys.2018.04.006

[34] LeCun Y, Bengio Y, Hinton G (2015). Deep learning. Nature.

[35] Chouhan SS, Singh UP, Jain S (2020). Applications of computer vision in plant pathology: a survey. Arch Comput Methods Eng.

[36] Lanjewar MG, Parab JS (2024). Cnn and transfer learning methods with augmentation for citrus leaf diseases detection using paas cloud on mobile. Multimed Tools Appl.

[37] Warne PP, Ganorkar S (2015). Detection of diseases on cotton leaves using k-mean clustering method. Int Res J Eng Technol (IRJET).

[38] Arivazhagan S, Ligi SV (2018). Mango leaf diseases identification using convolutional neural network. Int J Pure Appl Math.

[39] Lanjewar MG, Panchbhai KG (2023). Convolutional neural network based tea leaf disease prediction system on smart phone using paas cloud. Neural Comput Appl.

[40] Lu Y, Yi S, Zeng N, Liu Y, Zhang Y (2017). Identification of rice diseases using deep convolutional neural networks. Neurocomputing.

[41] Lanjewar MG, Morajkar PPP. Modified transfer learning frameworks to identify potato leaf diseases. Multimed Tools Appl. 83(17), 50401–50423 (2024) 10.1007/s11042-023-17610-0

[42] Amara J, Bouaziz B, Algergawy AA. Deep learning-based approach for banana leaf diseases classification. Datenbanksysteme für Business, Technologie und Web (BTW 2017)-Workshopband. 2017.

[43] Chaudhari V, Patil C (2014). Disease detection of cotton leaves using advanced image processing. Int J Adv Comput Res.

[44] Ahmed MR (2021). Leveraging convolutional neural network and transfer learning for cotton plant and leaf disease recognition. Int J Image Graph Signal Process.

[45] Singh V, Misra AK (2017). Detection of plant leaf diseases using image segmentation and soft computing techniques. Inf Process Agric.

[46] Kaur P, Harnal S, Tiwari R, Upadhyay S, Bhatia S, Mashat A, Alabdali AM (2022). Recognition of leaf disease using hybrid convolutional neural network by applying feature reduction. Sensors.

[47] Zhu W, Chen H, Ciechanowska I, Spaner D (2018). Application of infrared thermal imaging for the rapid diagnosis of crop disease. IFAC-PapersOnLine.

[48] Kumar M, Hazra T, Tripathy SS. Wheat leaf disease detection using image processing. Int J Latest Technol Eng Manag Appl Sci 6(4), 73–76 (2017)

[49] Liu B, Tan C, Li S, He J, Wang H (2020). A data augmentation method based on generative adversarial networks for grape leaf disease identification. IEEE Access.

[50] Debbal S, Bereksi-Reguig F (2007). Time-frequency analysis of the first and the second heartbeat sounds. Appl Math Comput.

[51] Nagaraju M, Chawla P, Upadhyay S, Tiwari R (2022). Convolution network model based leaf disease detection using augmentation techniques. Expert Syst.

[52] Li Z, Wu Z, He Y, Fulei C (2005). Hidden markov model-based fault diagnostics method in speed-up and speed-down process for rotating machinery. Mech Syst Signal Process.

[53] Mishra AM, Harnal S, Gautam V, Tiwari R, Upadhyay S (2022). Weed density estimation in soya bean crop using deep convolutional neural networks in smart agriculture. J Plant Dis Prot.

[54] Vallabhajosyula S, Sistla V, Kolli VKK (2022). Transfer learning-based deep ensemble neural network for plant leaf disease detection. J Plant Dis Prot.

[55] Maaten L, Huang G, Liu Z, Weinberger K. Densely connected convolutional networks. 2017. CVPR.

[56] He K, Zhang X, Ren S, Sun J Deep residual learning for image recognition. In: Proceedings of the IEEE Conference on Computer Vision and Pattern Recognition, 2016;770–778

[57] Sharma P, Berwal YPS, Ghai W (2020). Performance analysis of deep learning cnn models for disease detection in plants using image segmentation. Inf Process Agric.

[58] Huang G, Liu Z, Van DerMaaten L, Weinberger KQ. Densely connected convolutional networks. In: Proceedings of the IEEE Conference on Computer Vision and Pattern Recognition, 2017;4700–4708.

[59] Zhang S, Huang W, Zhang C (2019). Three-channel convolutional neural networks for vegetable leaf disease recognition. Cogn Syst Res.

[60] Barbedo JGA (2019). Plant disease identification from individual lesions and spots using deep learning. Biosyst Eng.

[61] Wu H, Wiesner-Hanks T, Stewart EL, DeChant C, Kaczmar N, Gore MA, Nelson RJ, Lipson H (2019). Autonomous detection of plant disease symptoms directly from aerial imagery. Plant Phenome J.

[62] Gandhi R, Nimbalkar S, Yelamanchili N, Ponkshe S. Plant disease detection using cnns and gans as an augmentative approach. In: 2018 IEEE International Conference on Innovative Research and Development (ICIRD), IEEE; 2018. pp. 1–5.

[63] Ma J, Du K, Zheng F, Zhang L, Gong Z, Sun Z (2018). A recognition method for cucumber diseases using leaf symptom images based on deep convolutional neural network. Comput Electron Agric.

[64] Chakravarthy AS, Raman S Early blight identification in tomato leaves using deep learning. In: 2020 International Conference on Contemporary Computing and Applications (IC3A), IEEE; 2020;154–158.

[65] Ramesh S, Vydeki D (2020). Recognition and classification of paddy leaf diseases using optimized deep neural network with jaya algorithm. Inf Process Agric.

[66] Li D, Wang R, Xie C, Liu L, Zhang J, Li R, Wang F, Zhou M, Liu W (2020). A recognition method for rice plant diseases and pests video detection based on deep convolutional neural network. Sensors.

[67] Caldeira RF, Santiago WE, Teruel B (2021). Identification of cotton leaf lesions using deep learning techniques. Sensors.

[68] Latif G, Abdelhamid SE, Mallouhy RE, Alghazo J, Kazimi ZA (2022). Deep learning utilization in agriculture: Detection of rice plant diseases using an improved cnn model. Plants.

[69] Ashwinkumar S, Rajagopal S, Manimaran V, Jegajothi B (2022). Automated plant leaf disease detection and classification using optimal mobilenet based convolutional neural networks. Mater Today Proc.

[70] Singh P, Singh P, Farooq U, Khurana SS, Verma JK, Kumar M. Cottonleafnet: cotton plant leaf disease detection using deep neural networks. Multimedia Tools and Applications. 82(24):37151–37176.

[71] Shrivastava A (2023). Cotton leaf and plant disease identification using intelligent deep learning technique. Int J Intell Syst Appl Eng.

[72] Peng ZK, Chu F (2004). Application of the wavelet transform in machine condition monitoring and fault diagnostics: a review with bibliography. Mech Syst Signal Process.

[73] Krizhevsky A, Sutskever I, Hinton GE (2017). Imagenet classification with deep convolutional neural networks. Commun ACM.

[74] Krizhevsky A, Sutskever I, Hinton GE. Imagenet classification with deep convolutional neural networks. In: Proceedings of the 25th International Conference on Neural Information Processing Systems—Volume 1. NIPS’12, pp. 1097–1105. Curran Associates Inc., 2012.

[75] Szegedy C, Liu W, Jia Y, Sermanet P, Reed S, Anguelov D, Erhan D, Vanhoucke V, Rabinovich A. Going deeper with convolutions. In: Proceedings of the IEEE Conference on Computer Vision and Pattern Recognition, pp. 1–9 (2015)

[76] Simonyan K, Zisserman A. Very deep convolutional networks for large-scale image recognition. CoRR abs/1409.1556 (2014)

[77] Szegedy C, Vanhoucke V, Ioffe S, Shlens J, Wojna Z. Rethinking the inception architecture for computer vision. In: 2016 IEEE Conference on Computer Vision and Pattern Recognition (CVPR), pp. 2818–2826 (2016). 10.1109/CVPR.2016.308

[78] He K, Zhang X, Ren S, Sun J. Delving Deep into rectifiers: surpassing human-level performance on ImageNet classification. 2015.

[79] Dietterich TG (2002). Ensemble learning. Handb Brain Theory Neural Netw.

[80] Dong X, Yu Z, Cao W, Shi Y, Ma Q (2020). A survey on ensemble learning. Front Comp Sci.

[81] Arpit D, Wang H, Zhou Y, Xiong C (2022). Ensemble of averages: improving model selection and boosting performance in domain generalization. Adv Neural Inf Proces Syst.

